# A protein–protein interaction inhibitor arrests the cell cycle in *Aspergillus fumigatus*

**DOI:** 10.1128/mbio.03563-25

**Published:** 2026-04-29

**Authors:** I. S. R. Storer, B. P. Thornton, A. S. Hackett, L. Tabernero, M. J. Bromley

**Affiliations:** 1 Manchester Fungal Infection Group (MFIG), Dept. Evolution Infection and Genomics, University of Manchester 5292, Manchester, United Kingdom; Universidade de Sao Paulo Campus de Ribeirao Preto, Ribeirao Preto, Sao Paulo, Brazil

**Keywords:** *Aspergillus fumigatus*, antifungal agents, cell cycle, mitosis, protein–protein interactions

## Abstract

Invasive infections caused by *Aspergillus fumigatus* have high mortality rates, even when treated with the first-line agent voriconazole. The global emergence of azole resistance further increases treatment failure, underscoring the urgent need for antifungals with novel mechanisms of action. The fungal cell cycle is essential for viability and represents an attractive but underexplored target. In *A. fumigatus*, progression from G2 to M phase requires interaction between the phosphatase NimT and cyclin-dependent kinase NimX. Here, we characterize the role of this interaction and its inhibition by 2-fluoro-4-hydroxybenzonitrile (compound 1), a small molecule that targets the human Cdc25B–Cdk2 interface. Using mutagenesis, we show that NimT residues Arg438 and Arg442 are critical for NimT–NimX binding and confirm they are essential for viability. A co-immunoprecipitation assay demonstrates that compound 1 disrupts the interaction, while live-cell imaging shows that this inhibition arrests cell cycle progression. Our findings provide mechanistic insight into fungal mitosis and highlight cell cycle regulators as promising antifungal drug targets.

**IMPORTANCE:**

Invasive aspergillosis has high mortality and limited treatment options, threatened by rising drug resistance. Targeting the fungal cell cycle represents an unexplored strategy for antifungal drug development. The dynamic interaction between NimT and NimX is critical to the fungal duplication cycle. Here, we show evidence that, unlike in *Schizosaccharomyces pombe*, relocation of NimT from the nucleus to the cytoplasm mid-interphase is the switching event that causes activation of NimX and allows the cell cycle to progress. We also show that disruption of the NimT–NimX interaction can be achieved using a reversible small-molecule inhibitor that arrests the fungal duplication cycle, highlighting mitotic regulators as promising antifungal drug targets.

## INTRODUCTION

The filamentous fungal pathogen *Aspergillus fumigatus* is the major etiological agent in invasive and chronic forms of aspergillosis (1). Even with appropriate antifungal therapy, invasive aspergillosis, the most rapidly progressing form of the disease, has a mortality rate of around 50% (2). Resistance to first-line triazole therapeutics has spread across the globe in part because of the widespread use of analogous azole compounds as crop protection products (3–6). Mortality in patients who are infected with a resistant isolate is increased by around 20% (7). The specter of azole-resistant *Aspergillus* has driven significant investment in the development of antifungal drugs with novel mechanisms of action. Two compounds, olorofim and fosmanogepix, have shown significant progress and are currently in phase 3 trials. However, fungicides that share their mechanism of action have been developed for crop protection (8, 9), and we have shown that one of these, ipflufenoquin, can select for isolates that are cross-resistant to olorofim (10). The limited development pipeline and emergence of resistance have prompted the WHO to call for further development of antifungals with novel mechanisms of action (https://www.who.int/publications/i/item/9789240105140).

The cell cycle is a fundamental process in all cells that undergo mitotic division and has been the focus of many drug discovery efforts, particularly for the treatment of cancers. In filamentous fungi, this process is more accurately termed the “duplication cycle,” reflecting the absence of cytokinesis (cell division) and the presence of multinucleated hyphal compartments (11). In *Aspergillus* spp., the dynamics of nuclear division are tightly regulated by iterative phosphorylation and dephosphorylation events controlled by the cyclin-dependent kinase (CDK) NimX (ortholog of human CDK2), the cyclin NimE (cyclin B ortholog), and a phosphatase NimT (CDC25 ortholog) (11–13). While there are four CDKs (1, 2, 4, and 6) and three CDC25s (A, B, and C) that regulate the human cell cycle, single, non-redundant enzymes orchestrate these events in *Aspergillus* species, making them particularly attractive targets for novel antifungals (14, 15).

The cell cycle has gained only modest attention for the development of antimicrobials. This is predominantly due to the high conservation between proteins that regulate the cell cycle in humans and in pathogens. However, in bacteria, inhibitors of cell division have shown potent and selective activity against multi-drug-resistant strains of *Staphylococcus aureus* at sub-µg/mL concentrations (16). The antifungal griseofulvin inhibits mitosis by disrupting microtubule assembly (17). It is often administered to children with dermatophyte scalp infections (18). Recently, a compound that inhibits the morphogenesis checkpoint kinase AnkA (Swe1 in yeast) was found to induce cell cycle arrest in both *A. fumigatus* and *Candida* species, highlighting this pathway as a viable source of potential antifungal targets (19).

We have previously identified two druggable pockets on NimT through virtual screening (20). One pocket is near the active site, while the other, away from the active site, coincides with the human CDC25/CDK protein–protein interaction site (21, 22). Small molecules that bind at the protein–protein interface have been explored as anticancer agents (23–25), suggesting that ligands that disrupt this protein–protein interaction site can arrest the cell cycle (26).

Here, we confirm the critical role of NimT in the cell cycle of *A. fumigatus* and identify two arginine residues that mediate the NimT–NimX interaction. We reveal that 2-fluoro-4-hydroxybenzonitrile (compound 1 [23]), a commercially available small molecule that binds to human CDC25B (23), perturbs the growth of *A. fumigatus* and prevents NimX from binding to NimT *in vitro*. Finally, using live-cell imaging, we show that compound 1 halts nuclear division in *A. fumigatus*. These results further our understanding of cell cycle dynamics and provide evidence for the utility of mitotic inhibitors in drug discovery.

## RESULTS

### NimT and NimX exhibit temporally distinct, reciprocal localization patterns throughout the duplication cycle

**Fig 1:**
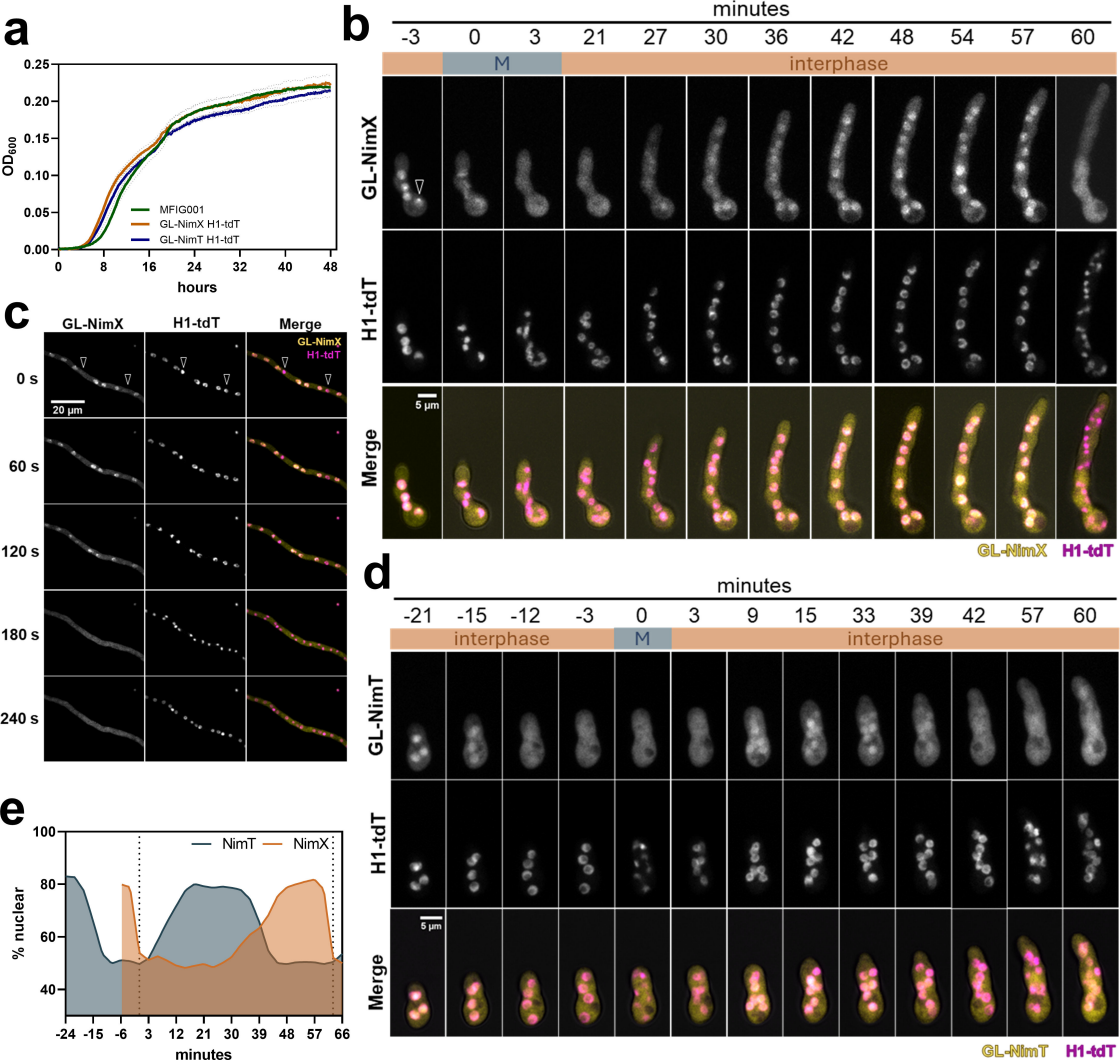
Dynamic localization of NimX and NimT during the duplication cycle in *A. fumigatus*. NimX localizes to the nucleus during late interphase and leaves the nucleus at mitosis, while NimT localizes to the nucleus during early interphase and is in the cytoplasm at mitosis (M). (**a**) Liquid growth assays of fluorescent protein fusion strains. Five microliters of a 1 × 10^5^ spores/mL solution of each strain was inoculated in liquid *Aspergillus* minimal media (AMM), pH 7. OD_600_ measurements were carried out during a 48 h period at 37°C. The total area under the curve (AUC) for MFIG001 was 6.983 (±0.023 SEM), for GL-NimT-H1-tdT was 6.787 (±0.019 SEM), and for GL-NimX-H1-tdT was 7.245 (±0.029 SEM). Differences in AUC were compared using a one-way ANOVA [F(2, 864) = 1.232, *P* = 0.292]. (**b**) mGL-NimX, H1-tdT germlings were imaged at 37°C in AMM. Images were captured every 3 min. NimX was visualized in the GL-NimX channel by capturing mGreenLantern-NimX fusion protein signal. The nucleus was visualized in the H1-tdT channel by capturing histone H1-tdTomato signal. Images are Z-stacked using average intensity projection. Time 0 indicates when mitosis starts. (**c**) Some nuclei do not accumulate NimX (open arrows) or enter mitosis. Images were captured in 1-min intervals. At 120 s, the mitotic wave can be seen in which nuclei nearest the tip apex enter mitosis first (condensing of H1), followed by nuclei further up the hyphae. Mitosis is completed in ~3 min. (**d**) mGL-NimT, H1-tdT germlings were imaged the same way as in panel **b**. (**e**) Overlay of mean nuclear percentage of NimT and NimX localization during the cell cycle. H1 signal was used to normalize NimT and NimX signals by condensation of the histones, indicating mitosis was occurring (dotted lines at minute 0 and minute 63). NimT is nuclear during early interphase and leaves the nucleus when NimX signal accumulates in the nucleus. At mitosis, NimT is in the cytoplasm, and NimX rapidly exits the nucleus. Because NimT or NimX is never excluded from the nucleus (i.e., there is never less NimT or NimX signal intensity in the nucleus than in the surrounding cytoplasm), the baseline nuclear signal is 50%*.*

Live-cell confocal imaging of germlings was used to understand the subcellular localization of NimT and NimX throughout the duplication cycle. We generated strains in which histone H1 (AFUB_042980) was tagged with tdTomato, and either NimT (AFUB_074160) or NimX (AFUB_073970) was tagged with mGreenLantern under the control of their native promoters. These fluorescent proteins were chosen as they had approximately equal brightness and did not have a large spectral overlap (27). The resulting strains, GL-NimT-H1-tdT and GL-NimX-H1-tdT, did not have any growth defects compared to the parental strain, MFIG001, when grown in liquid media (Fig. 1a).

NimX localization was monitored by live-cell fluorescent microscopy in 3-min intervals using the mGL-NimX, H1-tdT isolate (Fig. 1b). At *t* = −3 min (relative to mitosis), NimX appears predominantly in the nucleus at a focal point (indicated by an arrow in Fig. 1b) presumed to be spindle pole bodies based on comparative data from *Aspergillus nidulans* (28–30). At *t* = 0 min (mitosis), histone H1 appears to be condensing, and NimX is exiting the nucleus, suggesting nuclear division is in progress (prophase/metaphase). By minute 3, mitosis has occurred, and there is no evidence of NimX localization in the nucleus. During the following ~21 min, NimX does not co-localize with H1 in the nucleus but appears to be diffuse in the cytoplasm. By *t* = 27 min, NimX once again begins to co-localize with the nuclear H1 signal, and the signal gradually gains intensity in the nucleus up to *t* = 57 min, after which point mitosis occurs again (*t* = 60 min). The duplication cycle takes around 60 min ± 3 min (*n* = 10) in RPMI-1640 (Fig. S1). To gain more granular insights into NimX localization during mitosis, images were taken in 1-min intervals (Fig. 1c). Again, NimX appears to begin to leave the nucleus at early mitosis (120 s), and there is no nuclear NimX signal by late mitosis (180 s). This localization pattern mirrors that described for the NimX homolog in *Schizosaccharomyces pombe, Sp*CDC2, that accumulates in the nucleus during G2 and G2/M (31, 32). A mitotic wave is apparent at 120 s, in which the nuclei closest to the hyphal tip enter mitosis before the subapical nuclei, as seen by nuclear condensation. Furthermore, not all nuclei appear to accumulate NimX or enter mitosis (Fig. 1c, arrowheads).

There are currently no data on the temporal localization of NimT in any member of the *Aspergillus* species. To understand where NimT is localized throughout the cell cycle, the same assay was used as described for NimX, but using the mGL-NimT, H1-tdT isolate (Fig. 1d). NimT also cycled between the nucleus and cytoplasm but at different cell cycle phases when compared to NimX. At *t* = 0, the cell is in anaphase or telophase as nuclei have just replicated and appear condensed. NimT accumulates in the nucleus after mitosis by *t* = 9 min. Between *t* = 15 min and *t* = 33 min, the NimT signal remains predominantly nuclear. At *t* = 39 min, NimT begins to leave the nucleus and is cytoplasmic during mitosis at *t* = 57 min. This suggests that NimT has a nuclear role in early interphase, mirroring the role of CDC25A, one of the three isoforms that are homologous to NimT in vertebrates (33). Overlay analyses, using the point of nuclear division to synchronize data sets, show that NimT exits in the nucleus prior to NimX nuclear accumulation (Fig. 1e). The gradual nuclear import of NimX during late interphase suggests that the inactive, phosphorylated form of NimX is retained in the cytoplasm until it is activated by NimT, triggering nuclear import. Activation of CDK complexes by CDC25 in the cytoplasm as a prerequisite to CDK nuclear import has been demonstrated for human CDK1 (34, 35). These data suggest that the NimT relocation from nucleus to cytoplasm is the switching event that causes NimX activation. Interestingly, the localization pattern of *A. fumigatus* NimT is inverse to the shuttling pattern in *S. pombe* homolog CDC25, where *Sp*CDC25 concentrations peak in the nucleus at mitotic entry (G2/M) (32, 36). The conserved NimX/*Sp*CDC2 shuttling but opposing NimT/*Sp*CDC25 shuttling may reflect different cell cycle regulation requirements for coenocytic filamentous fungi.

### NimT and NimX are non-redundant and conserved interface residues that are critical for *A. fumigatus* viability

To consider the NimT–NimX interaction site as an antifungal target, we first wished to ensure that both gene products were essential for viability in *A. fumigatus.* We had indirect evidence of the essential nature of NimT, as only heterokaryons were generated from multiple knockout attempts (20). Similarly, NimX was identified as essential in *A. nidulans* using heterokaryon rescue (37).

**Fig 2:**
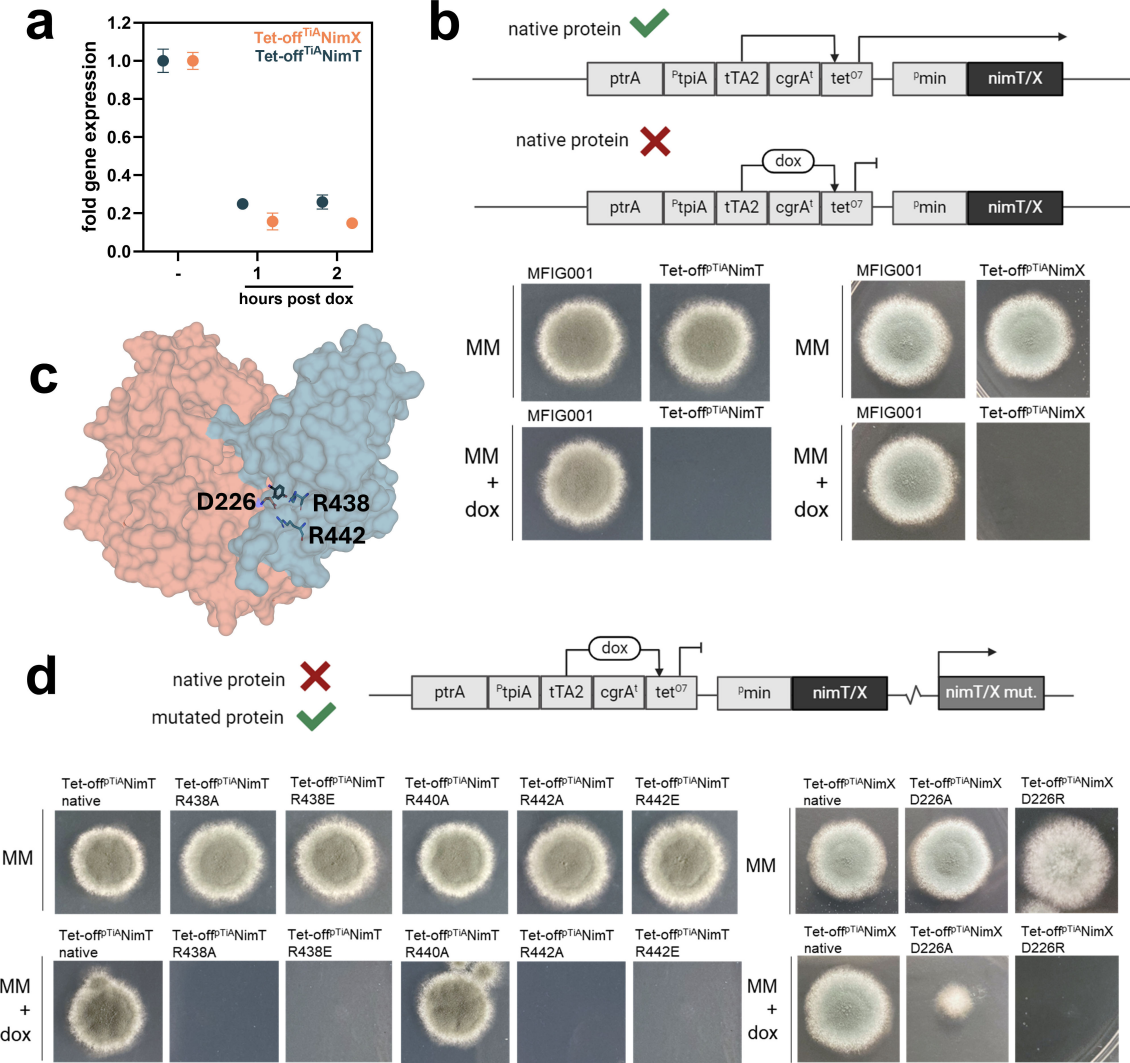
Site-directed mutagenesis of residues mediating the protein–protein interaction in NimT and NimX. (**a**) RT-qPCR of 18 h-old mycelia of Tet-off^pTiA^NimT and Tet-off^pTiA^NimX without (−) and 1- and 2-h post-addition of 1 mg/L dox. Expression is normalized to β-tubulin expression. Mean ± SEM of four technical replicates is shown. Post hoc analysis using Šidák’s correction indicated that both 1 h (mean 0.249, 0.014 SEM) and 2 h (mean 0.259, 0.037 SEM) of dox treatment led to significant (*P* < 0.001) reduction in NimT expression compared to no dox (mean 1.0, 0.061 SEM). Similarly, both 1 h (mean 0.157, 0.044 SEM) and 2 h (mean 0.148, 0.003 SEM) of dox treatment led to significant (*P* < 0.001) reduction in NimX expression compared to no dox (mean 1.0, 0.044 SEM). (**b**) Schematic of Tet-off cassette. The pyrithiamine cassette (ptrA) allows selection of transformants. The tpiA promoter (^P^tpiA) drives the transactivator (tTA2), which binds to the operator binding site (tet^O7^) and allows transcription of the gene by ^P^min. In the presence of doxycycline (dox), expression is inhibited by dox binding to tTA2, preventing its binding to tet^O7^. Tet-off^pTiA^NimT and Tet-off^pTiA^NimX are unable to grow in the presence of dox. One thousand spores were inoculated onto minimal media (MM) and MM with 10 mg/L doxycycline (MM + dox) and incubated for 48 h at 37°C. (**c**) NimX (orange) in complex with NimT (blue) modeled using AlphaFold-Multimer. The side chains of NimX D226 and NimT R438 and R442, and the predicted binding site of compound 1 (black) are shown. (**d**) Another copy of nimT or nimX, native or mutant allele, was inserted into the Aft4 region. Effects of these allele insertions can be seen when dox is added to suppress transcription of native nimT or nimX. NimT mutations at R438A, R438E, R442A, and R442E were not able to grow on MM + dox, whereas the R440A allele was. The NimX D226A mutation abolished growth, and D226R showed a reduction in growth on MM + dox*.*

To investigate the ability of *A. fumigatus* to grow with limited levels of NimT and NimX, strains were generated in which repressible promoters (Tet-off^pTiA^NimT and Tet-off^pTiA^NimX) replaced their native promoters. When transcription of each gene was repressed by the addition of doxycycline to the growth media (Fig. 2a), both strains were unable to grow (Fig. 2b), confirming that both NimT and NimX are essential, non-redundant proteins. Colony growth was restored to wild-type levels by ectopic expression of the native *nimT* or *nimX* from the *Aft4* safe-haven (38) locus in the Tet-off^pTiA^NimT and Tet-off^pTiA^NimX isolates.

Next, we explored whether the interaction between NimT and NimX was critical for the viability of *A. fumigatus*. Alignment of NimT to CDC25 and NimX to CDK2 revealed NimT Arg438 and Arg442, and NimX Asp226 were orthologous to sites that mediate the interaction between the human proteins (21, 39) (Fig. 2c). Using site-directed mutagenesis, we generated modified alleles of nimT and nimX: nimT^R438A^, nimT^R438E^, nimT^R442A^, nimT^R442E^, nimX^D226A^, and nimX^D226R^. These alleles were placed at the *Aft4* locus in Tet-off^pTiA^NimT or Tet-off^pTiA^NimX. The R438A, R438E, R442A, and R442E alleles were unable to complement the loss of nimT expression following the addition of doxycycline to growth media (Fig. 2d), demonstrating their critical role in NimT function. However, another allele modified at a site not associated with NimT/NimX interaction (R440A) was able to rescue growth arrest in the Tet-off^pTiA^NimT strain. Consistent with a role for these amino acids in cell cycle progression, strains with the NimT R438A and R442A alleles were able to germinate but did not undergo mitosis when grown for 12 h in the presence of doxycycline (Fig. S2).

The strain harboring the NimX D226R allele was unable to rescue doxycycline-induced growth arrest of the Tet-off^pTiA^NimX strain, indicating an essential role for the aspartate at this site (Fig. 2d). Interestingly, the D226A allele was able to partially rescue the growth phenotype, indicating that other amino acids are tolerated at this site.

### A cell cycle inhibitor inhibits fungal growth and disrupts metabolic activity of *A. fumigatus*

Compound 1, 2-fluoro-4-hydroxybenzonitrile, binds to human CDC25B at a pocket that includes orthologs of the conserved NimT Arg438 and Arg442 residues (23). As Arg438 and Arg442 are essential for viability, we performed a susceptibility assay of compound 1 on *A. fumigatus* to see if it was able to inhibit growth.

**Fig 3:**
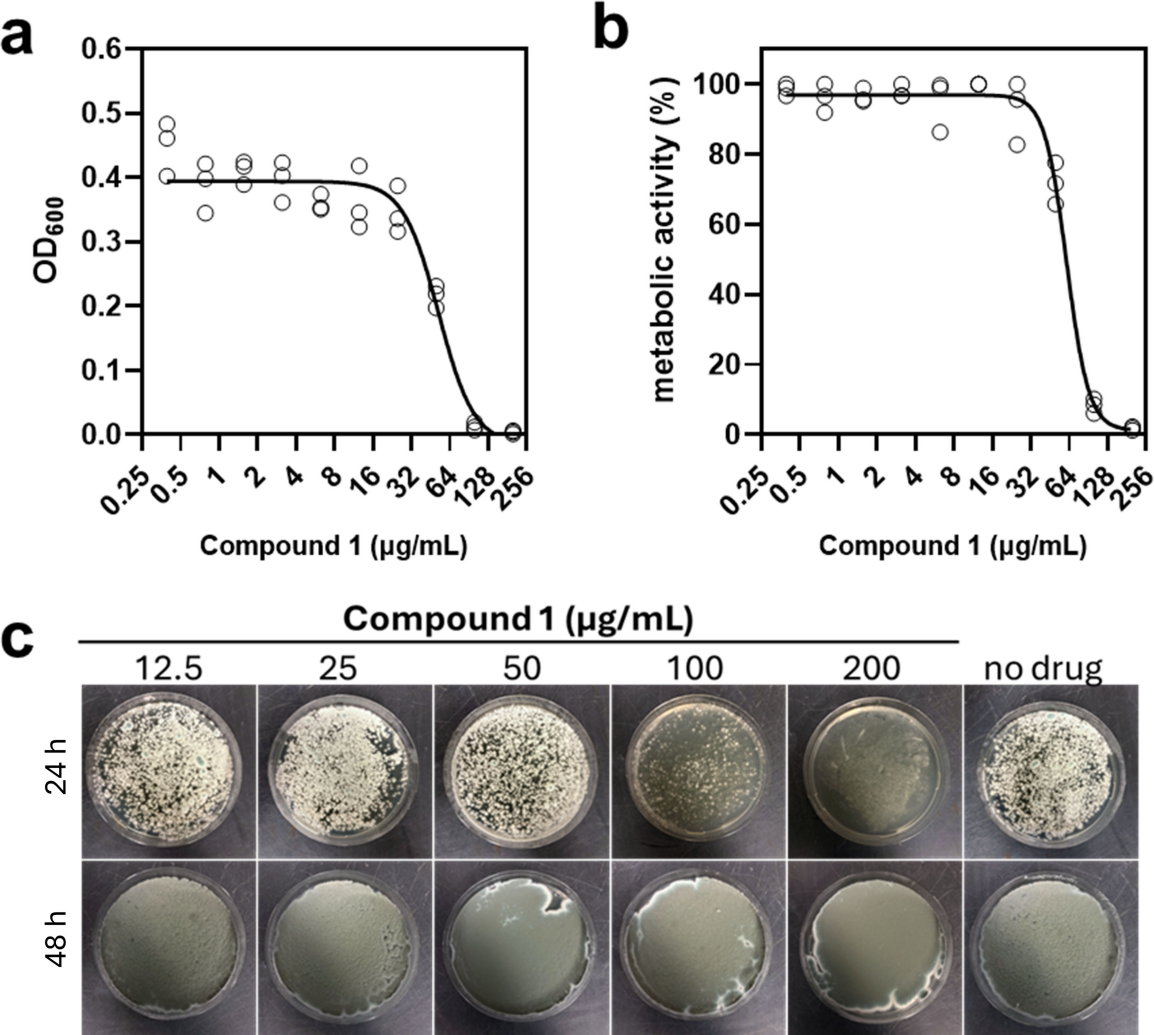
Activity of compound 1 on *A. fumigatus.* MFIG001 conidia were exposed to the indicated concentration of compound in RPMI-1640 for 48 h at 37°C. (**a**) MIC assay of compound 1 measured by OD_600_. (**b**) Modified MIC assay where metabolic activity was measured by the ability of cells to convert resazurin to the fluorescent product resorufin over 24 h. Metabolic activity was calculated as a percentage of the untreated control. Experiments are in biological triplicate. (**c**) Colonies derived from *A. fumigatus* conidia (MFIG001) pre-treated with compound 1 for 24 h at the indicated concentration and incubated at 37°C for 24 or 48 h in SAB.

Compound 1 inhibited the growth of *A. fumigatus* completely at 100 mg/L with an IC50 of 58.0 mg/L (Fig. 3a). A modified minimum inhibitory concentration (MIC), utilizing the metabolic dye resazurin, was used to determine if compound 1 inhibited metabolic activity in addition to growth (Fig. 3b). At 100 mg/L, the fungus retained 8.28% (±1.19 SEM) of the metabolic activity of the untreated control, consistent with cell cycle arrest but not cell death. To determine if compound 1 did indeed cause growth arrest but not death, we performed a killing assay. Spores were pre-treated with varying concentrations of compound 1 for 24 h in RPMI-1640 and then plated on SAB for 48 h to observe colony recovery (Fig. 3c). Recovery assays confirmed reversible growth inhibition, further supporting a fungistatic mechanism. After 24 h of outgrowth, colonies derived from spores treated with 100 and 200 mg/L of compound were smaller and less established than the untreated control; however, after 48 h, a lawn of growth consistent with untreated growth was observed for all compound 1 concentrations.

### NimT binds to NimX *in vitro*, but NimT R438 or R442 variant alleles and compound 1 prevent the interaction

**Fig 4:**
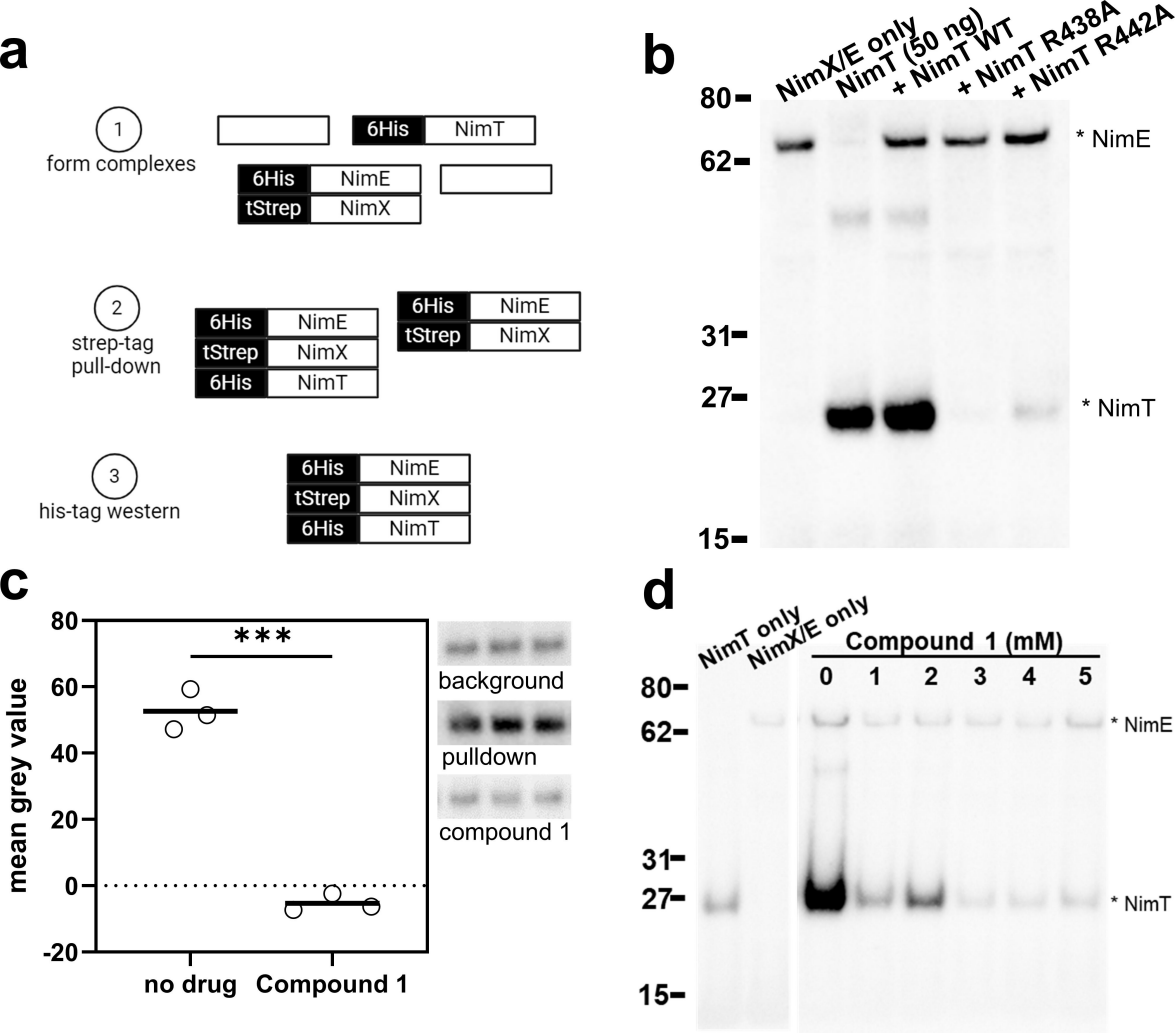
NimT–NimX co-immunoprecipitation assay. (**a**) Schematic of interaction assay. Step 1: NimX–NimE complexes are generated. His6-NimT is generated and purified and added to NimX complexes. Step 2: NimT that is associated with NimX is pulled down via the tStrep tag on NimX, and unbound proteins are washed away. Step 3: after washing, NimT–NimX interactions are detected by western blot to detect the His6 tag on NimT. (**b**) tStrep-tag purification of NimX and western blot analysis of NimT reveal that NimX associates with wild-type NimT, but not R438A or R442A NimT. (**c**) Western blot analysis of NimT recovered when 1 mM of compound 1 was added to NimT–NimX complexes. Values represent NimT band intensity quantified from western blots performed in triplicate. (**d**) Increasing concentrations of compound 1 do not decrease the amount of NimT binding to NimX.

To experimentally assess the physical interaction between NimT and NimX, an assay was developed in which tagged, recombinantly expressed active NimX^Str^NimE^His^ protein complexes would act as the “bait” to capture tagged NimT^His^ “prey” (see schematic Fig. 4a and Fig. S3). A western blot using an anti-His antibody was used to visualize any NimT co-purified with NimX^Str^ complexes (Fig. 4a). Using this co-immunoprecipitation method, we assessed the ability of wild-type, R438A, and R442A NimT proteins to bind to NimX (Fig. 4b). We were able to show reproducible recovery of the wild-type NimT protein with NimX complexes; however, R438A- and R442A-variant NimT proteins were not effectively co-precipitated (Fig. 4b).

To assess if compound 1 inhibited the NimT–NimX interaction *in vitro*, 1 mM (137.1 mg/L) of compound 1 was incubated with the proteins before co-precipitation. To account for incomplete removal of NimT during washing, a NimT-only control was used to provide a “background” signal (Fig. 4c). The intensity of the NimT band on the western blot was used to quantify the amount of NimT associated with NimX. NimX bound significantly less NimT with the addition of compound 1 [unpaired *t*-test, *t*(4) = 15.05, *P* <0.0001]. Higher concentrations did not decrease binding further (Fig. 4d). These findings indicate that compound 1 perturbs the NimT–NimX interface through orthosteric binding at a pocket containing Arg438 and Arg442.

To gain evidence that compounds could be developed that exhibit selective inhibition of the NimT–NimX interaction over their human counterparts, we tested a more potent CDC25-CDK2 inhibitor compound 7. Critically, we show that compound 1 is more potent than compound 7 at inhibiting the NimT–NimX interaction, suggesting that differences at the protein–protein interface could be exploited to develop selective compounds (Fig. S4a through d). In addition, we assessed compound 1 in a cytotoxicity assay using the A549 human epithelial cell line and were able to demonstrate that 200 mg/L (the equivalent of 2× the MIC in *A. fumigatus*) of compound 1 did not cause cytotoxicity following 24 h exposure (Fig. S4e).

### Compound 1, but not other antifungals, rapidly halts mitosis and causes NimT to mislocalize.

**Fig 5:**
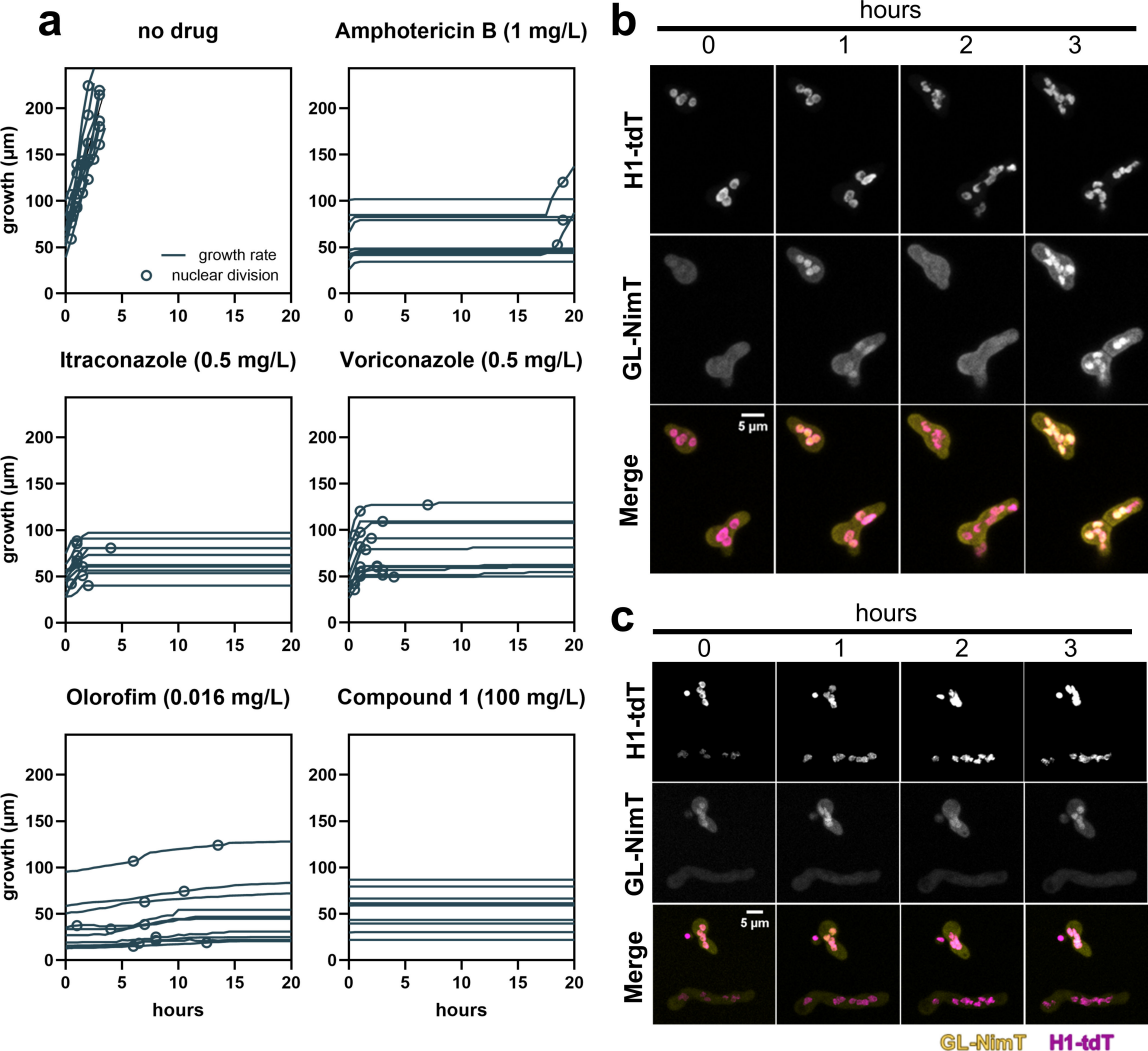
Nuclear divisions and growth rates of antifungal-treated germlings over 20 h. (**a**) Germlings were assessed for an increase in length (growth µm, solid lines) and nuclear divisions (circles) (*n* = 10). Measurements were not taken for the full 20-h period in the no drug control due to germlings growing multiple germ tubes or growing out of the microscope focus plane. Measurements were taken using ImageJ. Pre-grown mGL-NimT, H1-tdT germlings were treated with (**b**) 0.5 mg/L voriconazole or (**c**) 0.016 mg/L olorofim for 3 h. Images were acquired every hour to assess the number of nuclei and the localization of NimT.

We hypothesized that compound 1 would halt nuclear division, while antifungal agents targeting other cellular structures or proteins would not initially cause cell cycle arrest. To elucidate the effects of antifungals on the duplication cycle, H1-GFP tagged germlings, which had completed 2–3 cell cycles, were exposed to MIC concentrations of amphotericin B, itraconazole, voriconazole, olorofim, and compound 1. Germlings were treated for 20 h, and brightfield and H1-GFP images were taken every half hour to quantify germling growth and nuclear divisions (Fig. 5a). A representative germling is shown for each treatment in Fig. S5. Upon azole exposure, germlings increased in length for the first ~2 h following treatment, and multiple nuclear divisions occurred during this time. After 2 h, most germlings did not continue to grow. Amphotericin B appeared to halt growth within half an hour of exposure; however, some germlings resumed growth and nuclear divisions after ~18 h of exposure. Olorofim caused immediate inhibition of hyphal extension, but gradual swelling still occurred, consistent with previous observations (40). Germlings treated with olorofim underwent one or two divisions in 20 h. In contrast, compound 1 caused immediate arrest of hyphal extension, and no nuclear divisions occurred in the following 20 h.

**Fig 6:**
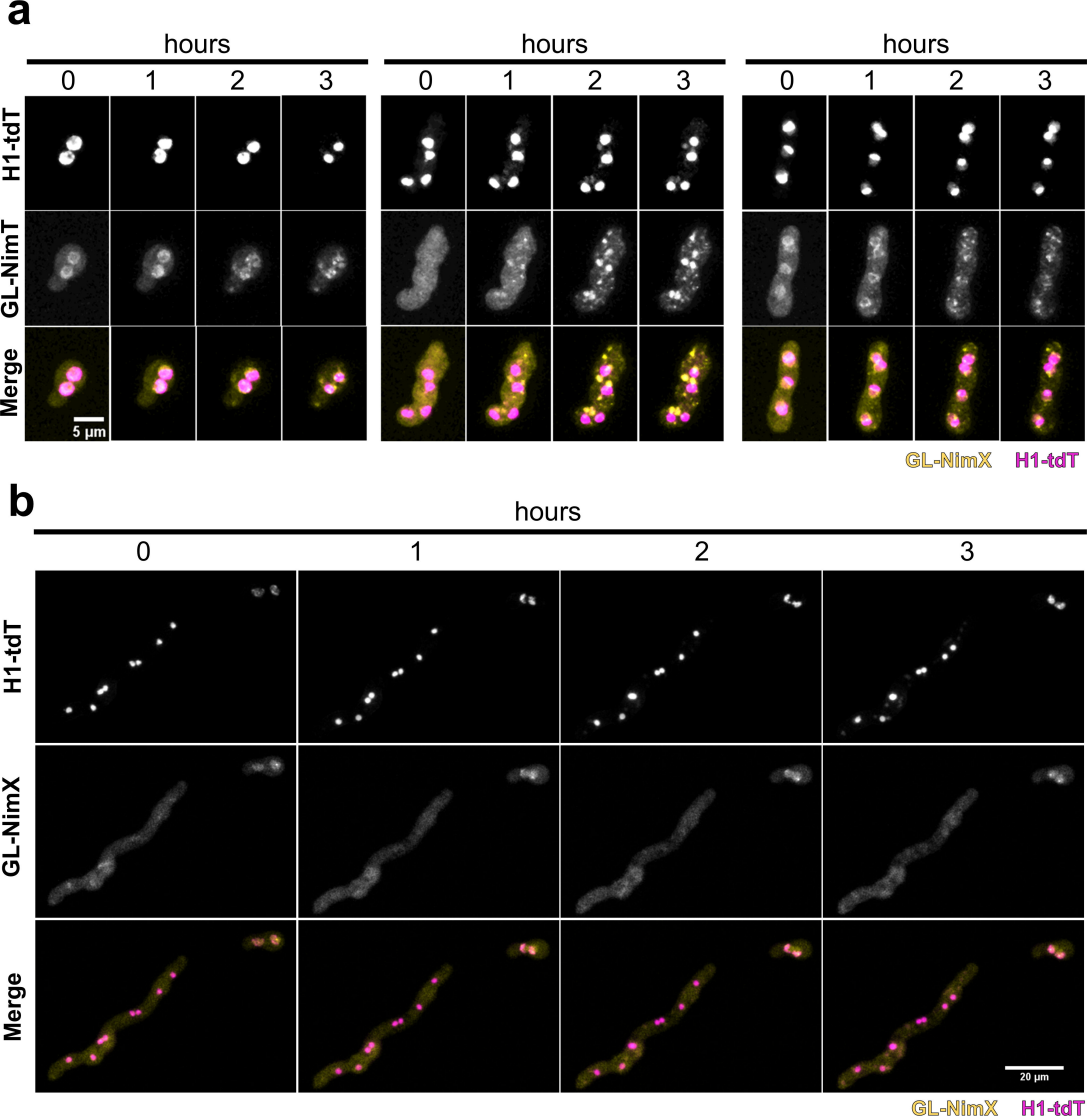
During treatment with compound 1, mitosis does not occur, and NimT, but not NimX, localizes to distinct foci. (**a**) Pre-grown germlings of mGL-NimT, H1-tdT were treated with 100 mg/L (MIC) of compound 1 and imaged over 3 h when three nuclear divisions would typically occur. NimT aggregates and does not cycle in and out of the nucleus. (**b**) Pre-grown germlings of mGL-NimX, H1-tdT were treated with 100 mg/L (MIC) of compound 1 and imaged during 3 h of treatment. NimX stays nuclear (top germling) and cytoplasmic (bottom germling) but does not cycle between, and mitosis does not occur.

To assess the role of antifungals on NimT cycling, mGL-NimT H1-tdT germlings were exposed to the drug, and cycling was observed over a 3-h period. Voriconazole caused growth arrest, but nuclear division and shuttling of NimT into and out of the nucleus continued (Fig. 5b, arrowhead). Olorofim, a pyrimidine biosynthesis inhibitor, caused cell cycle arrest, and no shuttling of NimT was observed (41) (Fig. 5c). As with olorofim, germlings exposed to compound 1 did not undergo mitosis, and NimT localization did not follow normal cycling patterns (Fig. 1d). However, in contrast to what was seen with olorofim, within 2 h, the NimT signal aggregated in foci throughout the cell. This localization abnormality seemed to take longer in cells in early interphase when NimT was in the nucleus (Fig. 6a: left and right germling) compared to later interphase when NimT was cytosolic (Fig. 6a: center germling). To discount the hypothesis that NimT could simply be aggregating due to a non-specific effect of compound 1 on all proteins, the localization of NimX over the same exposure duration was determined (Fig. 6b). Two germlings, where one appeared to be in late interphase due to the nuclear localization of NimX (upper germling) and one germling at G2/M or early G1 due to condensed appearance of H1 and NimX leaving the nucleus (lower germling), were followed. NimX signal remained in the same subcellular location throughout the observation period. NimX did not aggregate in distinct foci as NimT did, suggesting that the cell was not targeting it for degradation. This supports our hypothesis that NimT is being selectively targeted by compound 1.

## DISCUSSION

This work establishes the NimT–NimX protein–protein interaction as an essential mechanism in *A. fumigatus* cell cycle progression and identifies a small molecule, compound 1, that disrupts this interaction to induce mitotic arrest. These findings provide further insight into fungal mitosis and offer promise for the development of antifungal agents targeting mitosis.

Experimental data have indicated the importance of NimT and NimX for cell viability in other fungal species. NimT is essential in *S. pombe* (42), the filamentous fungus *Ustilago maydis* (43), and the model organism *A. nidulans* (44, 45). It appears to be dispensable for viability in *Candida albicans* (46–48). Interestingly, there is no NimT ortholog in plants (49), which may limit potential toxic off-target effects if similar inhibitors were to be used as fungicides in crop protection. Consistent with our data, NimX homologs are essential in all fungal species examined, including *C. albicans* (50), *S. pombe* (51), *U. maydis* (52), and *A. nidulans* (53). The localization patterns of NimT and NimX were determined throughout the cell cycle. The localization pattern of NimX has previously been described in *A. nidulans*, either by inference from the localization of the associated cyclin, NimE (29, 30), or by tagging NimX directly (28). Our observations are largely consistent with these data: NimX was predominantly cytoplasmic during the early interphase but accumulated in the nuclei as the interphase progressed. NimX was concentrated at the spindle pole bodies in the nucleus during the G2/M transition and dissipates during mitosis. Interestingly, there was a subset of nuclei in mature hyphae that did not accumulate NimX or enter mitosis. These nuclei are distinct from those in the subapical compartment, which do not enter the cell cycle (11, 54). Instead, they are in the growing tip, which usually undergoes synchronized mitosis (55). This phenomenon was described by Nayak et al. (28), who found that some nuclei do not accumulate cyclin B (NimE), which is physically associated with NimX. However, Nayak et al. concluded that this failure to accumulate NimE or enter mitosis was due to failed inactivation of the anaphase-promoting complex (APC) because of mutations in gamma-tubulin (MipA). They suggested that this failure of APC inactivation before the S phase caused NimE proteolysis. However, as far as we are aware, our strains do not harbor this mutation, which suggests other factors can trigger some nuclei to not re-enter the duplication cycle.

**Fig 7:**
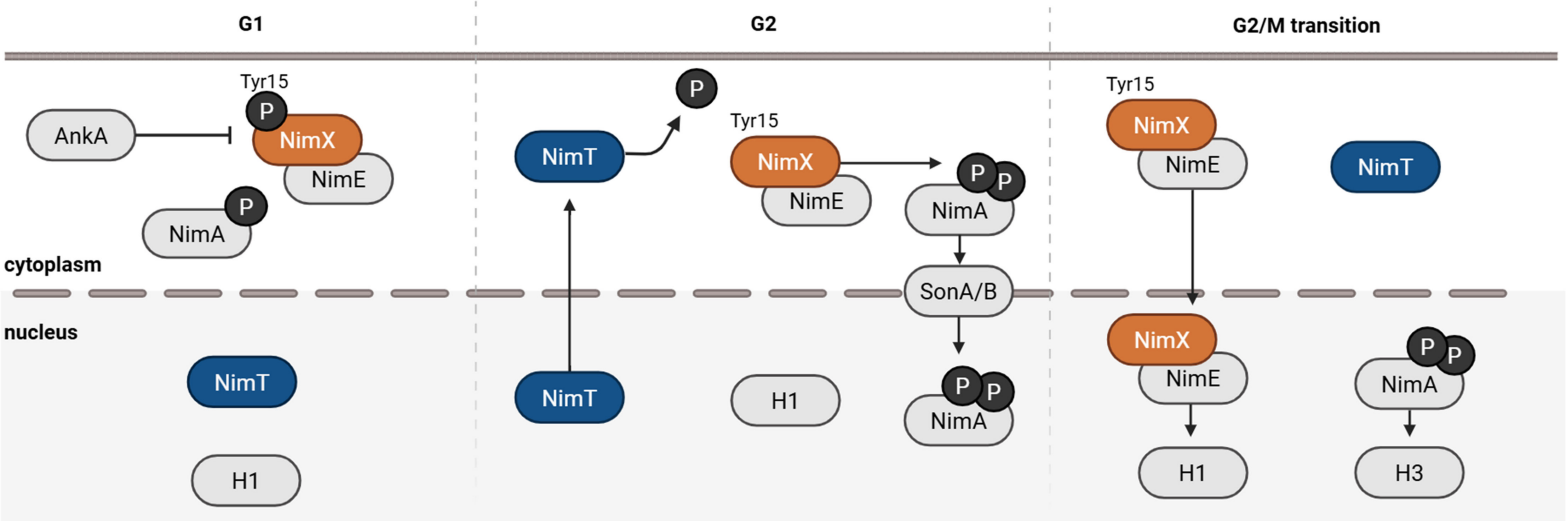
Proposed model of cell cycle progression in *A. fumigatus.* Localization of NimT (orange) and NimX (blue) during phases of the cell cycle. During G1, NimX associates with NimE. The complex is kept in its inactivated form by phosphorylation at Tyr15 by AnkA. NimA is partially activated by auto-phosphorylation or phosphorylation from another kinase. NimT is nuclear. During G2, NimT exits the nucleus. NimT activates NimX by dephosphorylation at Tyr15. Activated NimX hyper-phosphorylates NimA, which phosphorylates nuclear pore complex (NPC) proteins SonA and SonB, leading to NPC disassembly. At the G2/M transition, active NimX/NimE accumulates in the nucleus. NimX/NimE and NimA act upon multiple substrates to induce mitosis.

The localization of NimT has not been explored in *Aspergillus* species; however, NimT was assumed to cycle between the cytoplasm and nucleus in a similar manner to that seen for CDC25 in *S. pombe,* which accumulates in the nucleus during G2, reaching peak levels at mitosis (32). Our data indicate that the converse is true for NimT. During early interphase, NimT is predominantly in the nucleus and exits the nucleus during G2, remaining cytoplasmic during mitosis. These data suggest that NimT acts on substrates in the nucleus, possibly during the G1/S transition, and that the NimT–NimX interaction occurs afterward in the cytoplasm (Fig. 7). Determining the localization of NimT and NimX allowed us to assess the length of the cell cycle in *A. fumigatus*. Nuclear division occurred every 60 min, with mitosis occupying <3 min. This is somewhat longer than the previously documented times of 45 and 2.25 min, respectively (13). However, there are experimental discrepancies between the two studies that may account for this difference; for instance, the growth media we used and the *A. fumigatus* isolate differ from those of reference 13.

Although NimT has been shown to dephosphorylate NimX in *A. nidulans* (56), the direct interaction has not been experimentally confirmed. For the first time, this work provides experimental evidence for the interaction between NimT and NimX in *A. fumigatus*. Importantly, we show that recombinant NimX is unable to interact with NimT variants modified at residues Arg438 and Arg442 in *in vitro* protein assays, and these residues are essential for *A. fumigatus* growth and cell cycle progression. The detrimental effects of disrupting a single residue highlight the potential of this region as a targetable interface. It should be noted that our attempts to construct strains that carried both fluorescently tagged NimT and NimX variants were unsuccessful, possibly because the tags impaired complex formation, so we were unable to directly visualize the interaction *in vivo*. Nevertheless, our data support the physiological relevance of this interaction.

The NimT–NimX phosphatase-kinase interaction is conserved throughout eukaryotes to initiate mitosis (15, 57, 58). Co-crystal structures show that compound 1 binds to a pocket on CDC25B comprising residues required to bind CDK2 (23). Here, we demonstrate that compound 1 prevents NimT and NimX from forming a heteromeric complex, providing evidence that compound 1 binds at the site of Arg438 and Arg442. Treatment of germlings with compound 1 arrested the cell cycle. None of the treated germlings went through a nuclear division, suggesting that the mitosis-promoting NimT–NimX interaction was successfully inhibited by compound 1. This mechanism of action was distinct from amphotericin B, itraconazole, voriconazole, and olorofim. Notably, the action of compound 1 appeared to be fungistatic with nuclear division restarting once the compound had been removed. Furthermore, the compound will only be active in compartments that are undergoing nuclear division. Given that mitosis in filamentous fungi is restricted to actively growing tips (55), cell cycle inhibitors would also be expected to show fungistatic effects in mycelial growth phases. This compound could also provide a useful tool to investigate the cell cycle and its regulation further in *Aspergillus* species.

NimT localized to distinct foci with increasing intensity following treatment with compound 1. This localization is indicative of compound-NimT complexes being directed to proteasomes or lysosomes for degradation. This mislocalization of NimT supports our hypothesis that compound 1 is binding to NimT and perturbing its function. However, given the small molecular size of compound 1 and its lack of optimization, interactions with multiple cellular targets are possible. Therefore, the fungistatic effect may reflect a combination of on-target and off-target effects.

The homologous pocket in human CDC25B is smaller and only shares ~30% homology with the NimT pocket (20). Compound 1 shows very weak activity against the CDC25B (IC50 > 5 mM, [23]) and has no detectable toxicity in A549 cells at 2× MIC. Compound 1 is used here as a chemical probe to demonstrate the druggability of the NimT–NimX interaction interface rather than as a therapeutic lead. However, the defined binding pocket provides a clear opportunity for future structure-guided optimization. Solving the structure of NimT bound to compound 1 would reveal the protein–ligand interactions and guide the design of improved molecules to exploit interactions with unique residues in the fungal binding pocket. There is a possibility of generating *A. fumigatus*-specific inhibitors by increasing the number of ligand interactions with non-conserved amino acids.

Taken together, the results presented in this work have deepened our understanding of the role of NimT and NimX in the cell cycle and provided a strong rationale for the NimX-binding site of NimT as an antifungal drug target. The small molecule investigated in this work, compound 1, serves as a chemical probe demonstrating that disruption of the NimT–NimX interaction is sufficient to inhibit fungal growth and cell cycle progression.

## MATERIALS AND METHODS

### Strains, plasmids, and growth conditions used in this study

*A. fumigatus* strain MFIG001, derived from clinical isolate CEA10 lacking a functional *ku80* gene (59), was used as the parental isolate for genetic manipulations. *Escherichia coli* strain dh5α was used for general cloning, and strain C41(DE3) was used for protein expression. *A. fumigatus* strains and plasmids are listed in Table S1.

### Solid and liquid phenotyping

For growth curves, 500 conidia in 5 µL PBST were inoculated into 195 µL of Aspergillus minimal media (60) (AMM) in a CytoOne 96-well plate (Starlabs) and sealed with a Breathe-Easy sealing membrane (Sigma-Aldrich). Plates were incubated for 48 h at 37°C with OD_600_ measurements taken every 10 min using a Synergy 2 (BioTek) microplate reader. For phenotypic tests, 1,000 conidia in 5 µL PBST were inoculated onto Sabouraud dextrose agar (SAB). Plates were incubated for 48 h at 37°C before being photographed. Compound 1 (2-fluoro-4-hydroxybenzonitrile) was purchased from ThermoFisher Scientific.

### Strain generation

Transformations were either performed by homologous recombination following (61) or by CRISPR-Cas9-mediated transformation based upon (62). Briefly, *A. fumigatus* was grown overnight at 37°C in Sabouraud broth, followed by protoplasting using Vinotaste. Protoplasts were washed twice in 0.6 M KCl, followed by resuspension in 0.6 M KCl + 200 mM CaCl_2_. When used, guide RNA was formed by annealing crRNA to trans-activating CRISPR RNA (IDT) and ribonucleoproteins were formed by incubating at room temperature for 5 min with purified SpCas9 (IDT). Templates for transformation were generated as follows.

For strains used for live-cell imaging, mGreenLantern-tagged NimT or NimX was amplified from pUC19-mGreenLantern-NimT-ptrA or pUC19-mGreenLantern-NimX-ptrA and was used to replace the native nimT or nimX gene by CRISPR-Cas9. Fusion PCR (61) was used to insert the tdTomato sequence from ptdTomato and the *hph* cassette from pAN7.1 downstream of histone H1 (AFUB_042980) by homologous recombination to generate GL-NimT-H1-tdT and GL-NimX-H1-tdT. Transformants were selected for on YPS media (20 g/L yeast extract, 0.6 g/L Tris base, 5 g/L peptone, 342 g/L sucrose, 15 g/L agar, pH 6) supplemented with 200 mg/L hygromycin B (Enzo). To generate promoter replacement strains, the Tet-off^pTiA^ cassette was amplified from pSK606, and transformation was carried out as described in reference 62 to generate Tet-off^pTiA^NimT and Tet-off^pTiA^NimX. Mutated alleles of NimT and NimX were generated by site-directed mutagenesis performed on pUC19-nimT and pUC19-nimX, where overlapping primers were designed to change the wild-type codon to a different codon (63). Tet-off^pTiA^NimT and Tet-off^pTiA^NimX were used as the parental strains, where mutated and native versions of nimT and nimX from pUC19-nimTR438A, pUC19-nimTR438E, pUC19-nimTR440A, pUC19-nimTR442A, pUC19-nimTR442E, pUC19-nimXD226A, and pUC19-nimXD226R replaced the *Aft4* locus. Transformants were selected for on MM with 1 M D-sorbitol supplemented with 0.1 mg/L pyrithiamine (Sigma-Aldrich).

### Modeling of the NimT–NimX complex and docking

The sequences of NimT (D333-V499) and full-length NimX were uploaded to ColabFold, an open-source platform (https://github.com/sokrypton/ColabFold), and the AlphaFold2-Multimer_v3 model was used with three recycles and one seed. For docking, a model of NimT (D333-V499) was generated via ColabFold. The AlphaFold2_ptm model was used with 20 recycles and one seed. VSpipe (64), a semi-automated pipeline, was used for docking compound 1 and compound 7 to NimT using Autodock Vina. The binding position of each compound at the lowest binding affinity (∆G) was visualized using PyMOL 2.5. Compounds were investigated for non-covalent interactions between the ligand and NimT using the following prediction cutoffs: <4 Å between carbons for hydrophobic interactions, <4 Å between acceptor and donor for hydrogen bonds, <4 Å between acceptor and donor for halogen bonds, and <5.5 Å between charge centers for salt bridges.

### Synthesis of 2-((2-cyano-3-fluoro-5-hydroxyphenyl)thio)ethane-1-sulfonic acid (compound 7)

Following an adapted procedure from that of reference 23. 2,6-Difluoro-4-hydroxybenzonitrile (500 mg, 3.22 mmol, 1 equiv.), DIPEA (620 µL, 3.55 mmol, 1.1 equiv.), MOMCl (270 µL, 3.55 mmol, 1.1 equiv.) and DCM (10 mL) were added to a round-bottom flask. The resulting mixture was then stirred at room temperature overnight. The residue was then filtered through silica and eluted with DCM. The solvent was removed under vacuum. To the resulting residue was then added sodium 2-mercaptoethane-1-sulfonate (530 mg, 3.22 mmol, 1 equiv.), potassium carbonate (890 mg, 6.44 mmol, 2 equiv.), and DMF (4 mL). The resulting mixture was then stirred at 100°C for 3 days. The solvent was then removed under vacuum. The residue was redissolved in water, and concentrated HCl aq. (ca. 3 mL) was added. The resulting mixture was stirred for 30 min. The solvent was again removed, and the residue was purified by formic acid modified reverse-phase chromatography to yield the title compound (310 mg, off-white solid, 29%). Analytical data are consistent with those of reference 23. 1H NMR (500 MHz, DMSO) δ 11.43 (bs, 1H), 6.69 (d, J = 2.1 Hz, 1H), 6.58 (dd, J = 11.4, 2.0 Hz, 1H), 3.27–3.18 (m, 2H), 2.78–2.68 (m, 2H).

### Fluorescence microscopy image acquisition

Germlings were grown by inoculating 1× 10^4^ spores in 200 µL in a µ-Slide 8-well high glass bottom chamber (Ibidi) at 30°C for 16 h. Germlings were moved to 37°C for at least 1 h prior to imaging experiments. RPMI-1640 was routinely used for localization experiments and to investigate antifungal effects on germlings. The duplication cycle length was determined in RPMI-1640, MM, and *Aspergillus* complete medium. Fluorescence microscopy images were captured with a fully motorized Leica SP8x laser scanning confocal microscope equipped with a 40×/0.85 NA HCX PL APO dry objective or a 63×/1.4 NA HC PL APO CS2 oil objective. Imaging was performed at 37°C. All images were captured in 8-bit at 1,040 × 1,040 pixels. Fluorescent proteins were excited using a white light laser at 20%. Excitation and emission for mGreenLantern and tdTomato were 503/514 nm and 554/581 nm, respectively. The fluorescence signal was captured in a 20 nm bandwidth spanning the maximum emission. For imaging 20-h antifungal exposures and 12-h doxycycline exposures, widefield microscopy was used. Fluorescence microscopy images were captured with an inverted Nikon Eclipse TE2000E microscope equipped with a 40× Plan Fluor (0.6 NA) dry objective. Images were acquired with a 12-bit Hamamatsu DXM1200F camera (Hamamatsu). Imaging was performed at 37°C. A 470 nm LED laser line (CoolLED) was used to excite GFP for 300 ms at 20%. Signal was captured using 525 ± 25 nm emission bandpass filters. For live-cell imaging, images were acquired 30 min apart for 12 or 20 h. All microscopy was processed in ImageJ 1.54. For Fig. 2, individual *Z* slices were checked to ensure that nuclear exclusion of NimT or NimX did not occur, and average intensity projections were obtained from *Z*-stack images collected through the entire germling volume.

### Reverse transcription-quantitative polymerase chain reaction

RT-qPCR was carried out using a Luna Universal One-Step RT-qPCR Kit as per the manufacturer’s instructions. Amplicon lengths were 100–200 bp and primers were designed to anneal at an exon-exon junction to prevent amplification from contaminating DNA. The following primers were used to amplify probes: TGCTTGGTGGTCGTCAGTAC and GTGCCGAGAAGCCTGAAGAT (NimX), ACAAGAGAGAAAGCGTCCGG and TTGACTTTCCGGTGCTGGTT (NimT), CTCCGTTCCTGAGTTGACCC and CACGGAAAATGGCAGAGCAG (TubA). Gene expression was quantified using the 2^−ΔΔCt^ method. Statistical significance was determined using a one-way ANOVA with Šidák’s corrections for multiple comparisons (GraphPad 9.3.1).

### MIC assay and modified MIC assay

MIC determination for compound 1 was carried out according to the methods outlined by EUCAST (EUCAST Definitive Document E.DEF 9.4). Briefly, 100 µL of 2–5 × 10^5^ cfu/mL MFIG001 conidia in distilled water supplemented with 0.1% Tween 20 was prepared. Compounds in DMSO were aliquoted in micro-dilution plates at 2× final concentrations in 2× RPMI-1640 (with L-glutamine and a pH indicator, without bicarbonate) supplemented with glucose to a final concentration of 2%. Conidia suspension was added to the wells. Plates were incubated for 48 h at 37°C. Growth was measured by OD_600_ using a Synergy 2 (BioTek) microplate reader. For the modified MIC assay, after 24 h incubation, resazurin (Merck, UK) was added to each well to a final concentration of 44 µM and incubated for a further 24 h at 37°C. The ability of metabolically active cells to convert resazurin to the fluorescent product resorufin was quantified with a CLARIOstar plate reader (BMG Labtech, UK) using an excitation wavelength of 530 nm and an emission wavelength of 590 nm. For the killing assay, the MIC was set up as previously, but after 24 h of treatment, the well contents were washed in PBS and plated onto SAB. Plates were incubated for 48 h at 37°C.

### Protein expression and purification

pNIC28-NimT encodes a his6-tagged D333-K504 construct. NimT was expressed for 24 h at 18°C via IPTG induction. Cells were harvested and lysed by sonication in lysis buffer (PBS, 1% Triton X-100, 1 mg/mL lysozyme, cOmplete, Mini, EDTA-free Protease Inhibitor Cocktail [Merck], pH 7.4). NimT was purified from cleared lysates using NEBExpress Ni Spin Columns (NEB). NimT concentration was quantified using a Nanodrop 2000c spectrophotometer (ThermoFisher Scientific) with UV adsorption at 280 nm. The extinction coefficient was 20,280 M^−1^ cm^−1^, and the molecular weight was 22.74 kDa.

NimX complexes were generated by coexpression, as detailed in Fig. S3. pET151-NimE and pRSFDuet-AnkA were codon-optimized for expression in *E. coli* and were synthesized by ThermoFisher. pET151-NimE encoded a 6his-tagged full-length NimE (AFUB_069140) sequence. pRSFDuet-AnkA encoded an untagged AnkA (AFUB_023730) kinase domain sequence F704-V1046 with a start codon before F704. pCDF-NimX-CAK was generated by seamless cloning. pCDFDuet-CDKA-Cak1 was linearized by PCR (Phusion Flash Master Mix, ThermoFisher Scientific), omitting the unwanted CDKA gene and GST tag. NimX was amplified from pNimX and replaced the CDKA gene and GST tag, generating pCDF-NimX-CAK. pCDF-NimX-CAK, pET151-NimE, and pRSFDuet-AnkA were expressed for 24 h at 18°C via IPTG induction. Cells were harvested and lysed by sonication in lysis buffer (PBS, 2% Triton X-100, 1 mg/mL lysozyme, cOmplete, Mini, EDTA-free Protease Inhibitor Cocktail [Merck], pH 7.4). Cleared cell lysates were purified using NEBExpress Ni Spin Columns (NEB) using wash buffer consisting of PBS with 2% Triton X-100 and elution buffer consisting of PBS with 0.1% Triton X-100.

### Co-immunoprecipitation assay

Two hundred microliters of NimX/NimE complexes was mixed with 100 μg purified NimT and mixed using an end-over-end rotator at 4°C for 1 h. The supernatant was discarded, and 50 µL MagStrep Strep-Tactin Beads (IBA) were added, and complexes were captured using end-over-end rotation at 4°C for 1 h. The supernatant was removed, and 500 µL of wash buffer (PBS + 1% Triton X-100) was added to the beads and washed on an end-over-end rotator at 4°C for 18 h. The supernatant was discarded, and the washing step was repeated once more for 1 h. Thirty microliters of Laemmli SDS sample buffer, reducing (ThermoFisher Scientific), was added to the beads and heated to 99°C for 5 min to release the protein complexes. Samples were run on an SDS-PAGE gel, transferred to a PVDF membrane, and probed with an HRP anti-6× His tag antibody (Abcam). Band intensity was quantified in ImageJ 1.54.

### Lactate dehydrogenase assay

Lactate dehydrogenase release was quantified using a CytoTox 96 Non-Radioactive Cytotoxicity Assay (Promega). A549 cells were seeded at a density of 10,000 cells/well in a 96-well plate and left to adhere for 18 h at 37°C with 5% CO_2_. Cells were treated with the indicated amount of compound in DMSO diluted in RPMI-1640 (Merck) and incubated for 24 h at 37°C with 5% CO_2_. The CytoTox 96 assay was carried out per the manufacturer’s instructions. MIC values were calculated by EUCAST MIC determination for compound 1 (100 mg/L [0.73 mM]) or by the NimT–NimX interaction assay results for compound 7 (5 mM [1,386 mg/L]).

## SUPPLEMENTAL MATERIAL

Figure S1 mbio.03563-25-s0001.tif Duplication times in different media.

Figure S2 mbio.03563-25-s0002.tif NimT and NimX are required for cells to enter mitosis.

Figure S3 mbio.03563-25-s0003.tif Generation of active NimX complexes.

Figure S4 mbio.03563-25-s0004.tif Compound 7 inhibits the NimT-NimX interaction less effectively than compound 1.

Figure S5 mbio.03563-25-s0005.tif The duplication cycle in germlings continues during early treatment with antifungal drugs but halts when exposed to compound 1.

Table S1 mbio.03563-25-s0006.docx *A. fumigatus* strains and plasmids used in this work.

ASM does not own the copyrights to Supplemental Material that may be linked to, or accessed through, an article. The authors have granted ASM a non-exclusive, world-wide license to publish the Supplemental Material files. Please contact the corresponding author directly for reuse.
