# Supplementary figures and images for: A protein–protein interaction inhibitor arrests the cell cycle in Aspergillus fumigatus
Source: mBio. 2026 Apr 29;17(6):e03563-25. doi: 10.1128/mbio.03563-25 (PMC13251371; doi:10.1128/mbio.03563-25)

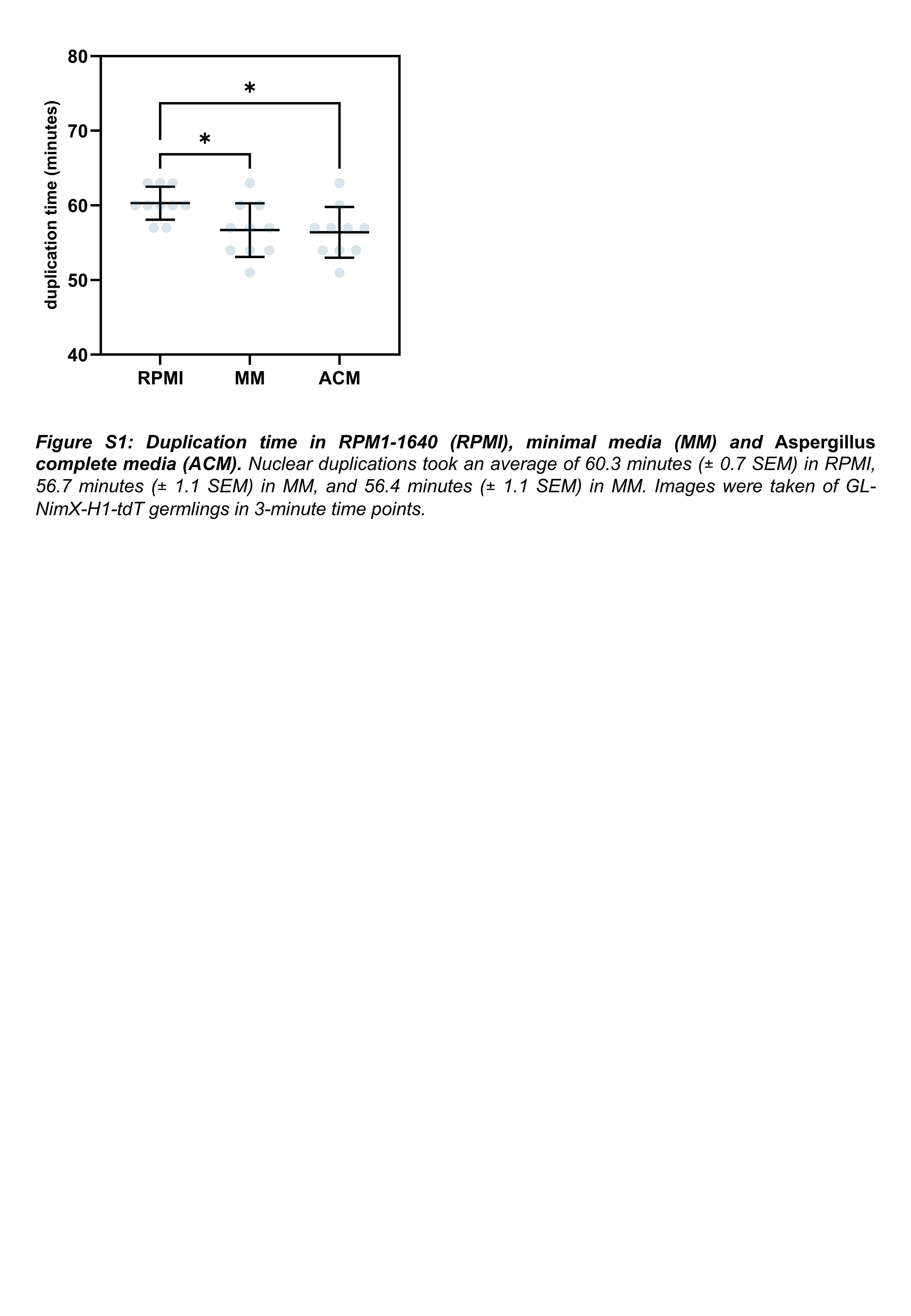

Supplement: Figure S1 — Duplication times in different media. [file mbio.03563-25-s0001.tif]

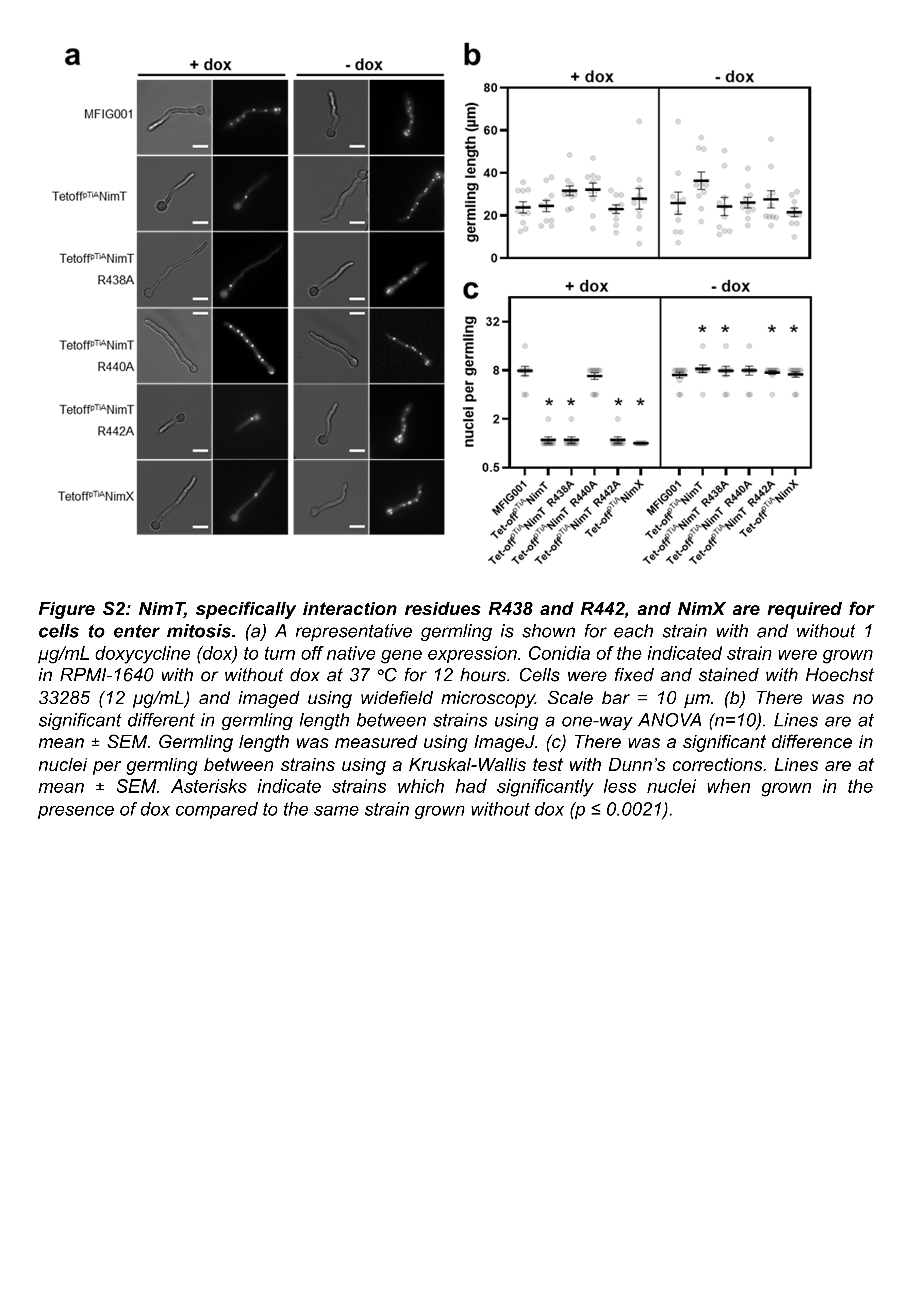

Supplement: Figure S2 — NimT and NimX are required for cells to enter mitosis. [file mbio.03563-25-s0002.tif]

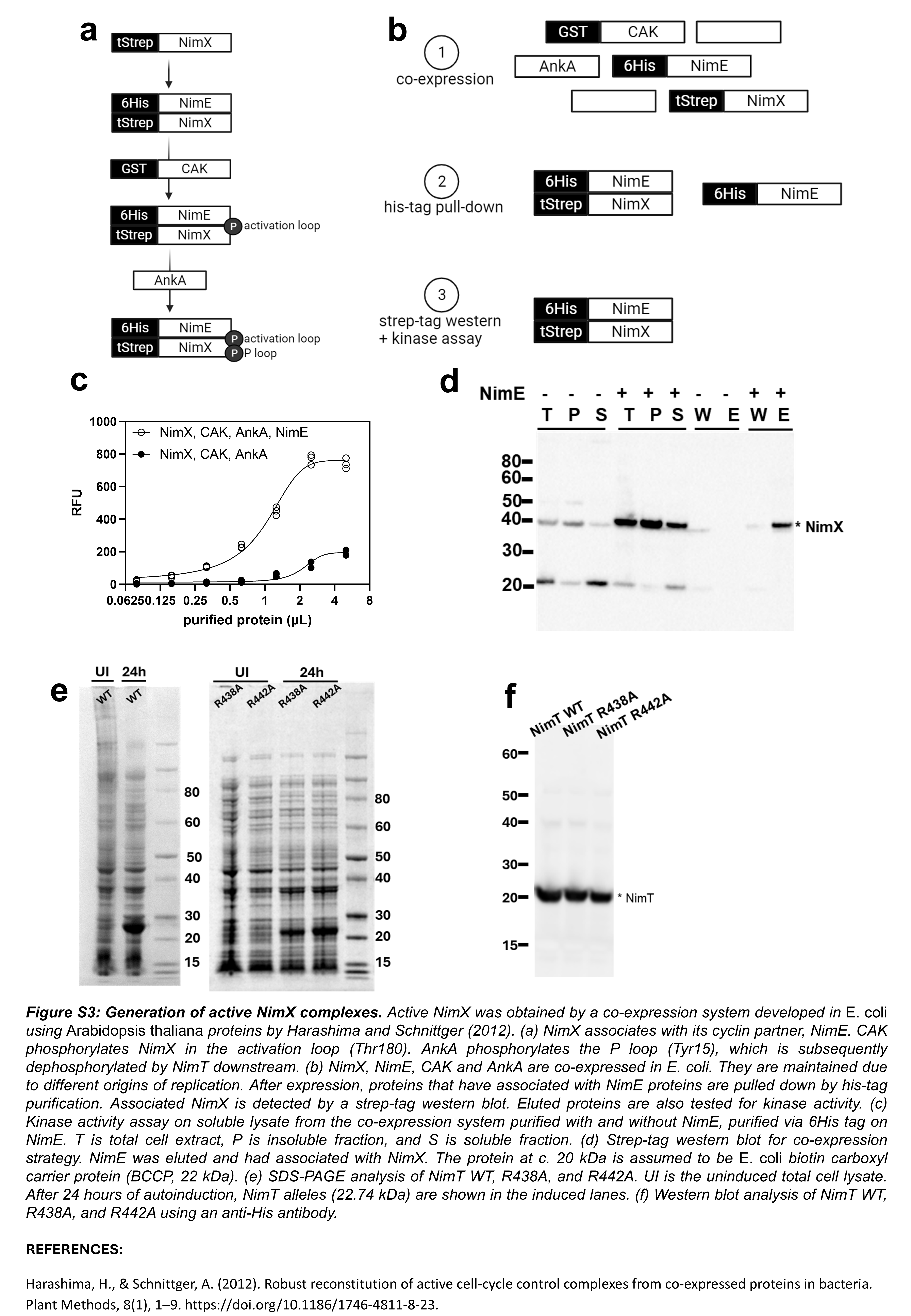

Supplement: Figure S3 — Generation of active NimX complexes. [file mbio.03563-25-s0003.tif]

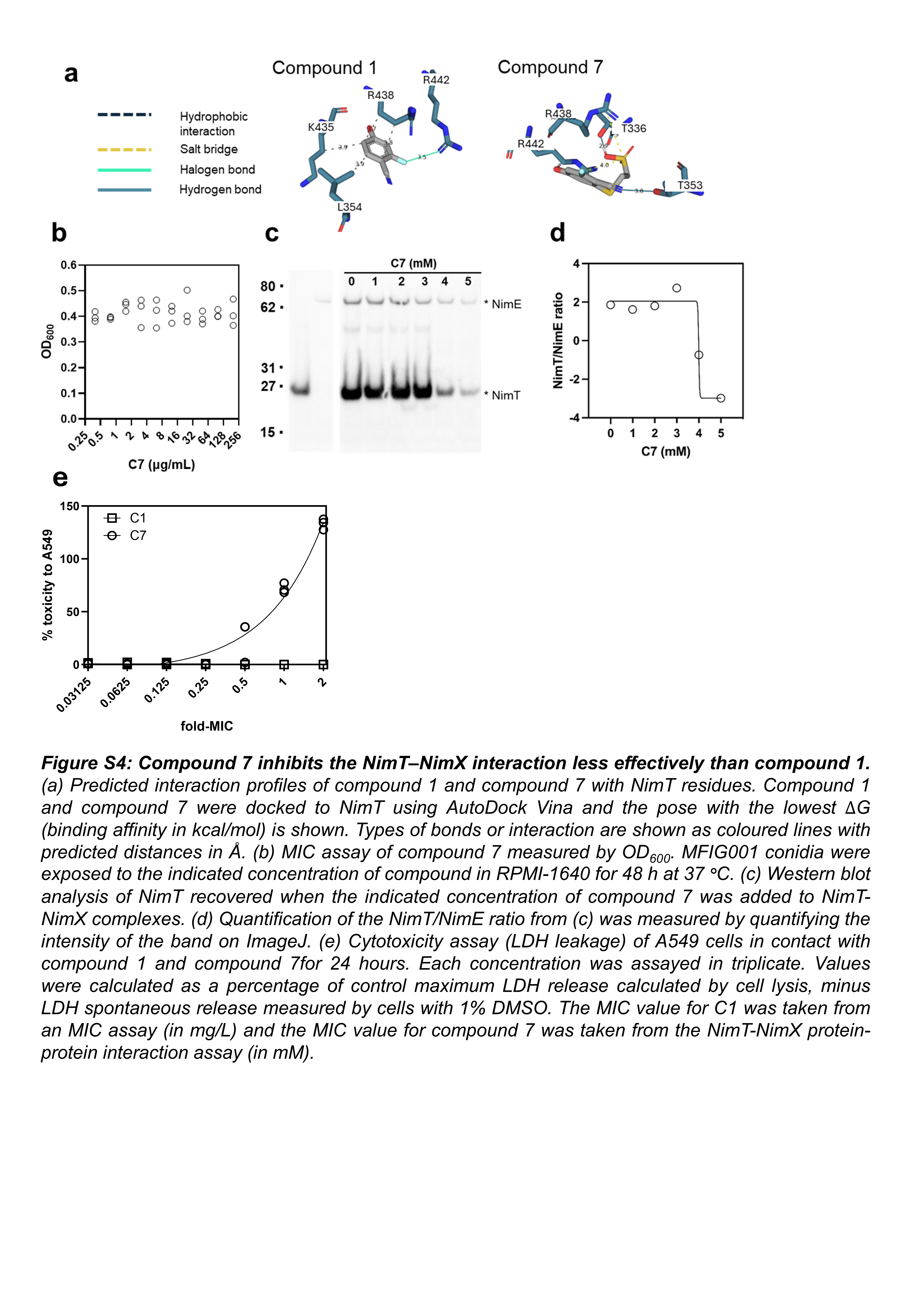

Supplement: Figure S4 — Compound 7 inhibits the NimT-NimX interaction less effectively than compound 1. [file mbio.03563-25-s0004.tif]

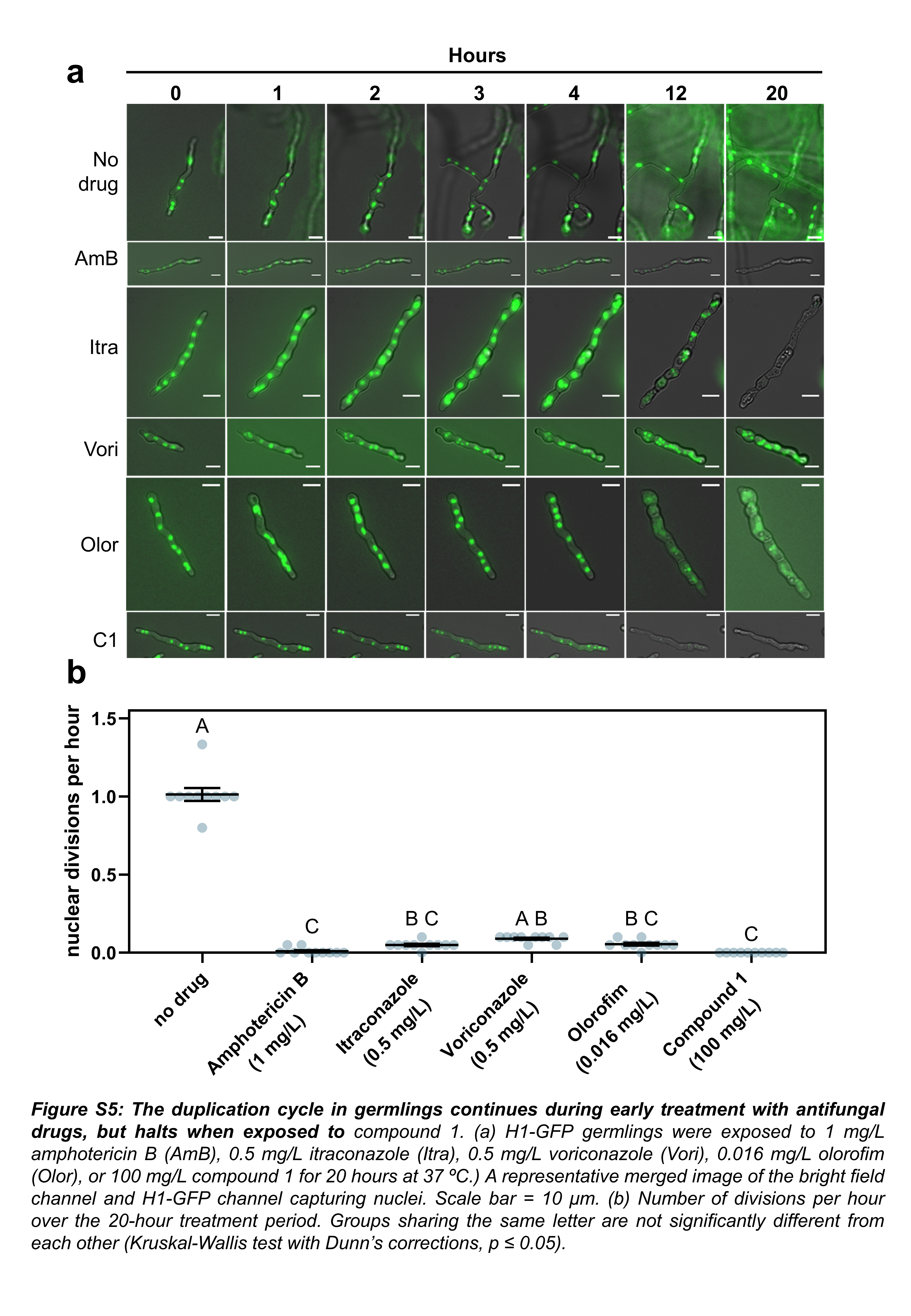

Supplement: Figure S5 — The duplication cycle in germlings continues during early treatment with antifungal drugs but halts when exposed to compound 1. [file mbio.03563-25-s0005.tif]
