# Supplementary material for: A protein–protein interaction inhibitor arrests the cell cycle in Aspergillus fumigatus
Source: mBio. 2026 Apr 29;17(6):e03563-25. doi: 10.1128/mbio.03563-25 (PMC13251371; doi:10.1128/mbio.03563-25)
Supplement: Table S1 — A. fumigatus strains and plasmids used in this work. [file mbio.03563-25-s0006.docx]

Supplementary table 1: A. fumigatus strains and plasmids, used in this work.

| ***A. fumigatus* strains** | | |
| --- | --- | --- |
| **Strain** | **Genotype** | **Source** |
| MFIG001 | wild type (A1160P+) | MFIG collection |
| Tet-off^pTiA^NimX | ΔPnimX:: Ptet0, ptrA | this work |
| Tet-off^pTiA^NimT | ΔPnimT:: Ptet0, ptrA | this work |
| Tet-offp^TiA^NimTnative | ΔPnimT:: Ptet0, ptrA, Δaft4::nimT | this work |
| Tet-offp^TiA^NimTR438A | ΔPnimT:: Ptet0, ptrA, Δaft4::nimTR438A | this work |
| Tet-offp^TiA^NimT R438E | ΔPnimT:: Ptet0, ptrA, Δaft4::nimTR438E | this work |
| Tet-offp^TiA^NimT R440A | ΔPnimT:: Ptet0, ptrA, Δaft4::nimTR440A | this work |
| Tet-offp^TiA^NimT R442A | ΔPnimT:: Ptet0, ptrA, Δaft4::nimTR442A | this work |
| Tet-offp^TiA^NimT R442E | ΔPnimT:: Ptet0, ptrA, Δaft4::nimTR442E | this work |
| Tet-offp^TiA^NimXnative | ΔPnimX:: Ptet0, ptrA, Δaft4::nimX | this work |
| Tet-offp^TiA^NimXD226A | ΔPnimX:: Ptet0, ptrA, Δaft4::nimXD226A | this work |
| Tet-offp^TiA^NimXD226R | ΔPnimX:: Ptet0, ptrA, Δaft4::nimXD226R | this work |
| GL-NimT-H1-tdT | ΔnimT∷mGreenLantern- nimT -ptrA, H1-tdT-hph | this work |
| GL-NimX-H1-tdT | ΔnimX∷mGreenLantern- nimX -ptrA, H1-tdT-hph | this work |
| H1-GFP | histone H1-GFP | MFIG collection |
| **Plasmids** | | |
| **Plasmid** | **Description** | **Source** |
| pNimX | Full length coding sequence of NimX codon optimised for *E. coli* | ThermoFisher |
| pNIC28-NimT | Expression of his6-tagged D333-K504, WT | this work |
| pNIC28-NimT^R438A^ | Expression of his6-tagged D333-K504, R438A | this work |
| pNIC28-NimT^R442A^ | Expression of his6-tagged D333-K504, R442A | this work |
| pET151-NimE | Expression of 6his-tagged full-length NimE | ThermoFisher |
| pRSFDuet-AnkA | untagged AnkA F704-V1046 | ThermoFisher |
| (67) | a kind gift from Dr. Arp Schnittger (University of Hamburg, Germany) | Harashima and Schnittger, 2012 |
| pCDF-NimX-CAK | Expression of full-length NimX and CAK | this work |
| pAN-7.1 | MFIG collection | Ridder and Osiewacz, 1992 |
| pSK606 | MFIG collection | Wanka et al., 2016 |
| pUC19-nimT | Full length Af coding sequence of NimT | this work |
| pUC19-nimX | Full length Af coding sequence of NimX | this work |
| pUC19-nimTR438A | Template for Tet-offp^TiA^NimTR438A | this work |
| pUC19-nimTR438E | Template for Tet-offp^TiA^NimT R438E | this work |
| pUC19-nimTR440A | Template for Tet-offp^TiA^NimT R440A | this work |
| pUC19-nimTR442A | Template for Tet-offp^TiA^NimT R442A | this work |
| pUC19-nimTR442E | Template for Tet-offp^TiA^NimT R442E | this work |
| pUC19-nimXD226A | Template for Tet-offp^TiA^NimXD226A | this work |
| pUC19-nimXD226R | Template for Tet-offp^TiA^NimXD226R | this work |
| ptdTomato | Template for tdTomato H1 tagging | Ortiz et al., 2022 |
| pUC19-mGreenLantern-NimT-ptrA | Template for GL-NimT-H1-tdT | this work |
| pUC19-mGreenLantern-NimX-ptrA | Template for GL-NimX-H1-tdT | this work |

**REFERENCES:**

Harashima, H., & Schnittger, A. (2012). Robust reconstitution of active cell-cycle control complexes from co-expressed proteins in bacteria. Plant Methods, 8(1), 1–9. https://doi.org/10.1186/1746-4811-8-23.

Ridder, R., & Osiewacz, H. D. (1992). Sequence analysis of the gene coding for glyceraldehyde-3-phosphate dehydrogenase (gpd) of Podospora anserina: use of homologous regulatory sequences to improve transformation efficiency. Current Genetics, 21(3), 207–213. https://doi.org/10.1007/BF00336843

Wanka, F., Cairns, T., Boecker, S., Berens, C., Happel, A., Zheng, X., Sun, J., Krappmann, S., & Meyer, V. (2016). Tet-on, or Tet-off, that is the question: Advanced conditional gene expression in Aspergillus. Fungal Genetics and Biology, 89. https://doi.org/10.1016/j.fgb.2015.11.003

Ortiz, S. C., Pennington, K., Thomson, D. D., & Bertuzzi, M. (2022). Novel Insights into Aspergillus fumigatus Pathogenesis and Host Response from State-of-the-Art Imaging of Host–Pathogen Interactions during Infection. Journal of Fungi, 8(3). https://doi.org/10.3390/JOF8030264
